# Cumulative keyboard strokes: a possible risk factor for carpal tunnel syndrome

**DOI:** 10.1186/1745-6673-7-16

**Published:** 2012-08-02

**Authors:** Andreas Eleftheriou, George Rachiotis, Socratis E Varitimidis, Charilaos Koutis, Konstantinos N Malizos, Christos Hadjichristodoulou1

**Affiliations:** 1 Department of Hygiene and Epidemiology, Faculty of Medicine, School of Health Sciences, University of Thessaly, 22, Papakyriazi str, Larissa, Thessaly, 41222, Greece; 2 Department of Orthopaedics and & Musculoskeletal Trauma Surgery, Faculty of Medicine, School of Health Sciences, University of Thessalia, Larissa, Greece; 3 Department of Public Health Technological Institute of Athens, Athens, Greece

## Abstract

**Background:**

Contradictory reports have been published regarding the association of Carpal Tunnel Syndrome (CTS) and the use of computer keyboard. Previous studies did not take into account the cumulative exposure to keyboard strokes among computer workers. The aim of the present study was to investigate the association between cumulative keyboard use (keyboard strokes) and CTS.

**Methods:**

Employees (461) from a Governmental data entry & processing unit agreed to participate (response rate: 84.1 %) in a cross-sectional study. Α questionnaire was distributed to the participants to obtain information on socio-demographics and risk factors for CTS. The participants were examined for signs and symptoms related to CTS and were asked if they had previous history or surgery for CTS. The cumulative amount of the keyboard strokes per worker per year was calculated by the use of payroll’s registry. Two case definitions for CTS were used. The first included subjects with personal history/surgery for CTS while the second included subjects that belonged to the first case definition plus those participants were identified through clinical examination.

**Results:**

Multivariate analysis used for both case definitions, indicated that those employees with high cumulative exposure to keyboard strokes were at increased risk of CTS (case definition A: OR = 2.23;95 % CI = 1.09-4.52 and case definition B: OR = 2.41; 95%CI = 1.36-4.25). A dose response pattern between cumulative exposure to keyboard strokes and CTS has been revealed (p < 0.001).

**Conclusions:**

The present study indicated a possible association between cumulative exposure to keyboard strokes and development of CTS. Cumulative exposure to key-board strokes would be taken into account as an exposure indicator regarding exposure assessment of computer workers. Further research is needed in order to test the results of the current study and assess causality between cumulative keyboard strokes and development of CT.

## Background

Carpal Tunnel Syndrome (CTS) is a clinical disorder resulting from the compression of the median nerve at the wrist. It has been considered as the most common of the entrapment neuropathies [1]. However a generally accepted case definition of this disorder has not been established, and thus a variety of case definitions for CTS have been used, especially in studies related to occupational risk factors for the development of the syndrome [2].Furthermore, contradictory reports have been published regarding the association of carpal tunnel syndrome and the use of computer keyboard. Two systematic reviews reported either that the balance of evidence did not indicate an important association between keyboard, and computer work and carpal tunnel syndrome and that there is insufficient epidemiological evidence that computer work causes CTS [3,4]. It has been stressed that there are several limitations related to the quality of the CTS case definition, and of exposure assessment used in the epidemiological studies on the occupational risk factors of CTS. Nevertheless, it has been stated that the question of whether intense keyboard use is associated with an increased or decreased risk of CTS is still unanswered [5].

We conducted a cross-sectional study of computer workers at a Governmental data entry& processing unit in order to investigate the possible impact of exposure to keyboard use on the development of Carpal Tunnel Syndrome through detailed measurement of the cumulative keyboard strokes.

## Subjects and methods

All 548 workers of a Governmental data entry & processing unit were invited to participate in the study. 461 accepted to participate in the study (response rate: 84.1 %).

Α questionnaire was distributed to the participants aiming to obtain information regarding sex, age, Body Mass Index (BMI), educational level, smoking habit, history of physical activity, and personal medical history: diabetes mellitus, rheumatoid arthritis, thyroid disorders and history of accidents. In particular, the ‘’physical activity” was measured by the use of a questionnaire. Participants asked to report the type and frequency (less than 1 day per month, 1–4 days per month, 1–2 days /per week and 3 or more days per week) of their physical activity (basketball, football, tennis, jogging, swimming). Participants were also asked if they had previous history of CTS or underwent surgery for CTS.

### Exposure assessment

The work schedule of the employees was 7.30 hours per day, for 5 days/ week. Employees took two brakes (duration of each brake: 15 min). The nature of keyboard work was related mainly to the insertion of numbers by the use of the right/left hand.

The assessment of workers exposure to keyboard use was performed by using the payroll records. The relevant information was available from the start of employment. It should be emphasized that the worker’s salary was keyboard stroke dependent. In particular, the amount of the keyboard stroke per worker per year has been calculated by the use of payroll records. The worker’s cumulative exposure to keyboard use was then calculated by multiplying the number of keyboard strokes per year by the years of work. Two categories of exposure (high exposure group, low exposure group) to keyboard use have been created and the cut-off level has been defined by the use of the ROC analysis [6]. An indicator variable with high discriminatory ability will have a curve with Area Under Curve (AUC) 0.5-1 and an indicator variable with low discriminatory ability will have an AUC ≤ 0.5.

Moreover, in order to assess a possible dose–response pattern between exposure to keyboard and CTS, four groups were formed based on percentiles of the exposure distribution among participants. In particular, subjects falling into the 25^th^ percentile (<101.400.000 key-board strokes-years) were considered as the very low exposure group. Participants falling into the 25^th^-50^th^ percentile of exposure (101.400.001-208.000.000 keyboards-strokes-years) were considered as the low exposure category. Workers that fell into the 50-75^th^ percentile (208.000.001-335.400.000 key-board strokes) were considered as the medium exposure category. Finally, employees falling into the 75^th^ -100 ^th^ percentile (>335.400.000 key-board-strokes-years) comprised the high exposure group.

### Outcome assessment

In order to synthesize the history of symptoms and the findings of clinical examination we used an approach based on the CTS-6 algorithm which has been developed by Graham through a Delphi consensus incorporating a variety of medical specialties [7]. The CTS-6 algorithm includes six dimensions: Two from the medical history (numbness predominately or exclusively in median nerve territory [3.5 points], and nocturnal numbness [4 points]. Four dimensions were related to physical examination: thenar atrophy and/or weakness [5 points]; positive Phalen test [5 points]; loss of two point discrimination [4.5 points]; and positive Tinel sign [4 points]. A score ≥12 is considered as very suggestive of a CTS diagnosis.

Participants completed a questionnaire on the previously mentioned CTS symptoms. Then, a clinical examination took place and focused on thenar atrophy and or weakness, Phalen’s test, Tinel’s test and loss of two point discrimination. The physician who performed the clinical examination was blinded to subject’s exposure status. In order to verify a possible association between cumulative exposure to keyboard use and CTS we used two case definitions for CTS.

#### Case definition A

Subjects with personal medical history of CTS or surgery due to CTS were considered as CTS cases. Subjects who reported surgery for CTS were examined by the physician in order to verify the existence of scar. Subjects who reported personal medical history of CTS (CTS diagnosed by an orthopedic surgeon) were asked to report confirmation of the diagnosis by the use of electro-diagnostic testing. In summary, case definition A was defined as CTS diagnosed by a physician (including surgery for CTS).

#### Case definition B

Subjects with score ≥ 12 (CTS-7 algorithm) or participants with a medical personal history of CTS or surgery for this disease were considered as CTS cases.

## Statistical analysis

Data collected were entered in a database created within Epi Info 2000 software. Absolute (n) and relative (%) frequencies were presented for qualitative variables, while quantitative (continuous) variables were presented as mean (standard deviation). Chi- square test or Fischer’s exact test was used for the univariate analysis of qualitative variables.

In order to identify risk factors independently associated with the development of carpal tunnel syndrome (two case definitions) two models of backward logistic regression analysis have been employed. In the first model the dependent variable was the case definition A and the independent variables included all risk factors that demonstrated statistical significance in the univariate analysis. In the second model the dependent variable was the Case definition B. In order to assess the correlation between age and exposure to keyboard use the Spearman correlation coefficient has been calculated. Chi-square test for trend was used for the assessment of a dose–response relationship between cumulative exposure to keyboard and CTS. Statistical analysis was conducted by the use of Epi- Info software and SPSS version 14.0. Relative Risk (RR) for univariate analysis and adjusted Odds Ratio (OR) for multivariate analysis and 95 % Confidence Interval (95 % CI) were calculated. The level of statistical significance was defined < 0.05. Moreover, because of the possible correlation between independent variables a second logistic regression model was employed regarding case definition B. In this model a p value ≤0.25 has been defined as a screening criterion (univariate analysis) for the inclusion of variables in logistic regression.

## Ethical approval

Ethical approval was obtained from the scientific committee of the University of Thessaly.

## Results

The study population was predominantly female (83.6 % females; 16.6 % males). The mean age was 45.2 years (SD = 9.46) and the mean duration of employment with computer work was 16.2 years (SD = 9.05).The range of work years with computer was 1 to 38, and the mean of keyboard strokes per year was 23.700.000. Moreover, 42.3 % of the participants were ever-smokers. Personal medical history of Rheumatoid Arthritis, Diabetes Mellitus or Disorders of the Thyroid gland was reported by 13.2 % of the participants. The mean BMI was 20. 6 (SD = 3.77). Fifty one participants (11 %) reported a personal medical history of CTS or a surgery for Carpal Tunnel Syndrome. One hundred sixteen subjects (25 %) have recorded significant clinical evidence of CTS (score in CTS algorithm ≥12).

### Results of ROC analysis

The application of ROC for case definition A revealed that the AUC was 0.642 (95%CI: 0.563 - 0.721; p-value =0.001) and the cut-off point was set at 240,500,000 keyboard strokes/years. For the case definition B the AUC was 0.668 (95%CI: 0.616 - 0.720); p-value < 0.001) and the cut-off point was set at 149,500,000 keyboard strokes/years. These results indicate that both indicator variables have demonstrated high discriminatory ability.

### Descriptive and multivariate analysis for case definition A

The prevalence of CTS (case definition A) was estimated at 11 %. Table 1 illustrates the univariate analysis of personal medical history of CTS or surgery for CTS. Total cumulative exposure to keyboard use was significantly associated with the development of CTS (RR = 2.38;95%CI = 1.38-4.12).

**Table 1:** Univariate analysis of case definition A

**Risk factor**	**CTS case definition A**	**RR (95 % CI)**
	**n/total (%)**	
Age		
> = 45	31/233 (13.3)	1.38 (0.81-2.35)
< 45	20/208 (9.6)	
BMI		
**> =** 25	28/212 (13.2)	1.22 (0.72-2.06)
**<** 25	22/204 (10.8)	
Smoking		
Ever-smoker	28/181(15.5)	1.74 (1.02-2.93)*
Non- smoker	22/247 (8.9)	
Physical activity		
Yes	8/144 (5.6)	0.40 (0.19-0.84)
No	43/317 (13.6)	
Rheumatoid Arthritis		
Yes	2/8 (25.0)	2.32 (0.67-7.89)
No	49/453 (10.8)	
Diabetes Mellitus	0/11 (0)	NA
Yes	51/450 (11.3)	
No		
Hypothyroidism		
Yes	7/42 (16.7)	1.58 (0.76-3.29)
No	44/419 (10.5)	
Hyperthyroidism		
Yes	0/14 (0)	NA
No	51/447 (11.4)	
Total keyboard strokes-years		
> =240500000	31/174 (17.8)	2.38 (1.38-4.12)**
<240500000	18/241 (7.5)	

Risk factors and personal medical history of CTS or surgery for CTS. (Case definition A).

*P = 0.003;chi-square test.

** P = 0,001;chi-square test.

NA: Not applicable.

Backward logistic regression analysis indicated that cumulative exposure to keyboard use and smoking, and physical activity were independent predictors of the development of CTS (Table 2).

**Table 2:** Logistic regression of case definition A

**Risk factor**	**CTS**	
	**OR (95 % CI)**	**P value**
Total keyboard strokes- years		
> =240500000	2.23 (1.09 to 4.52)	0.026
< 240500000		
Age		
> =45	1.16 (0.53 to 2.55)	0.701
< 45		
Physical activity		
Yes	0.38 (0.16 to 0.87)	0.023
No		
Smoking		
Ever	1.99 (1.01 to 3.54)	0.043
Non smoker		

(Case definition A).

### Descriptive and multivariate analysis for case definition B

The prevalence of CTS was estimated at 36 % (n = 167). Table 3 shows the univariate analysis of CTS. According to these results female sex (RR = 3.41;95%CI = 1.83-6.37, age >45 years old (RR = 1.31;95%CI = 1.02-1.68), and cumulative exposure to keyboard use(RR = 2.6;95 % CI = 1.8-3.73) were variables associated with the development of the carpal tunnel syndrome. On the other hand the report of regular physical activity has been found as a protective factor against the development of CTS (RR = 0.72;95 % CI = 0.54-0.96). Smoking habit and personal medical history of Rheumatoid Arthritis, Diabetes Mellitus, and disorders of the Thyroid gland did not show a statistically significant association with the prevalence of CTS.

**Table 3:** Univariate analysis of case definition B

**Risk factor**	**CTS. Case definition B**	**RR (95 % CI)**
	**n/total (%)**	
Sex		
Female	156/381 (40.9 %)	3.41
Male	9/75 (12 %)	(1.83 – 6.37)*
Age		
> = 45 years	97/233 (41.6)	1.31
< 45 years	66/208 (31.7)	(1.02 to 1.68)**
BMI		
**> =** 25	22/55 (40.0)	1.07
**<** 25	134/361(37.1)	(0.75 to 1.52)
Physical activity		
Yes	45/144 (31.3)	0.72
No	89/206 (43.2)	(0.54 to 0.96)***
Smoking		
Ever-smoker	77/181 (42.5)	1.22
Non-smoker	86/247 (34.8)	(0.96 to 1.55)
Rheumatoid Arthritis		
Yes	4/8 (50.0)	1.38
No	163/453 (36.0)	(0.68 to 2.80)
Diabetes Mellitus		
Yes	5/11 (45.5)	1.26
No	162/450 (36.0)	(0.65 to 2.44)
Hypothyroidism		
Yes	14/42 (33.3)	0.91
No	153/419 (36.5)	(0.58 to 1.42)
Hyperthyroidism		
Yes	4/14 (28.6)	0.78
No	163/447 (36.5)	(0.33 to 1.80)
Cumulative keyboard strokes-years		
> =149500000	128/268 (47.8)	2.60
<149500000	27/147 (18.4)	(1.80 to 3.73) *

Risk factors related to CTS (Case definition B).

*P < 0.001;chi-square test.

**P = 0.031;chi-square test.

*** P = 0.002;chi-square test.

Logistic regression analysis has shown (Table 4) that employees with cumulative exposure to keyboard use ≥ 149.500.000 key-strokes- years (high exposure) presented a 2,4 fold increased risk of CTS (OR = 2. 41; 95%CI = 1.36-4.25) in comparison to the low exposure category (<149.500.000 key strokes). Moreover, female sex was identified as an independent predictor of CTS (OR = 4.08;95%CI = 1.51-11.04). As shown in Table 2 and Table 4 no statistical significant association was found between age and CTS while our results indicated a correlation between age and exposure to keyboard Spearman’s correlation coefficient = 0.463; p <0.001). It should be stated that the inclusion of the variables for the multivariate analysis was based on a screening criterion of p ≤ 0.05. After the use of a less restrictive screening criterion (p ≤ 0.25) exposure to keyboard use remained a significant risk factor for CTS (OR = 2.5;95%CI = 1.4-4.47). This also was the case for sex (OR = 4.7;95%CI = 1.58-13.93). Smoking appeared to be a risk factor for CTS (OR = 1.69;95 % CI = 1.03-2.76).A dose–response pattern has been found regarding cumulative exposure to key-board and the risk of CTS (Table 5). In particular, among employees classified as very low, low, medium, and high exposure group the prevalence of CTS was found to be 17.3 %,32.7 %, 45.5 % and 54.4 %, respectively (Chi square for trend; p < 0.001).

**Table 4:** Multivariate (logistic regression) analysis of risk factors related to CTS (Case definitionB)

**Risk factor**	**CTS**	
	**OR (95 % CI)**	**P value**
Total keyboard strokes- years		
> =149500000	2.41 (1.36 to 4.25)	0.002
< 149500000		
Sex		
Female	4.08 (1.51 to 11.04)	0.005
Male		
Physical activity		
Yes	0.72 (0.44 to 1.20)	0.217
No		
Age		
> =45	1.48 (0.90 to 2.43)	0.117
< 45		

**Table 5:** Dose–response relationship between cumulative exposure to key board use and CTS

**Cumulative exposure (key board-years)**	**CTS.**	**OR**
	**n/total (%)**	
High exposure		
≥335.400.001	56/103 (54.4)	5.69
		
Medium exposure		
208.000.001-335.400.000	46/101 (45.5)	4.00
Low exposure		
101.400.001-208.000.000	35/107 (32.7)	2.32
Very low exposure		
≤101.400.000	18/104 (17.3 %)	1.00 (ref)

## Discussion

In the present study we found that cumulative keyboard use was an independent predictor of Carpal Tunnel Syndrome (CTS) among employees working at a data processing unit. In addition, it is worth mentioning that with both case definitions used, the exposure to cumulative keyboard use remained an independent determinant of the development of CTS syndrome even after controlling for several confounding factors. In addition, a dose–response relationship between cumulative exposure to keyboard use and risk of CTS has been found. Thomsen et al in a systematic review evaluating eight epidemiological studies concluded that the epidemiological evidence of an association between computer use and development of CTS is inconsistent [4]. This conclusion is in line with a more recent systematic review conducted by van Rijm et al [8]. In addition, the studies on the association between computer work and CTS have been characterized by heterogeneity in terms of study design, definition of exposure and outcome assessment. Two illustrative examples of this heterogeneity are the studies of Ali et al and Atroshi et al.Ali and Sathiyasekaran [9] where in an Indian workplace based cross sectional study found that the prevalence of CTS (case definition based on clinical features) was 13.1 % and also that time of computer work was an independent risk factor for CTS (OR: 2.5 to 4.9).On the contrary, Atroshi and coworkers in a large population study (case definition based on both physical examination and Nerve Conduction Tests) have reported a statistically significant protective effect of keyboard use (PR = 0.55;95%CI = 0.32-0.96) [10]. However, these two studies have a different approach in terms of exposure assessment in comparison to the present study. Both studies used questionnaires for the exposure assessment and didn’t take into account the cumulative exposure to keyboard strokes. In particular, in the study of Atroshi et al the participants were asked about the average daily time spent at a keyboard. A different approach for exposure assessment has been adopted by Ali et al. Participants were asked to report cumulative years of computer work, and daily hours of computer work per day. However, a distinction between exposure to keyboard and mouse use has not been made in this study.Multivariate analysis of our results has also shown that apart from occupational exposure to keyboard use, smoking status (ever-smokers) was an independent risk factors for CTS. This finding is in agreement with the results of other studies [10-12]. Our results have also revealed that regular physical activity was a protective factor against the development of CTS. It has been suggested that being physically inactive is a major risk factor for slowing and clinical CTS [13]. We are not able to provide an explanation for the possible protective role of physical activity on CTS, and future research is needed to further investigate this issue.Another finding which deserves to be discussed is the absence in multivariate analysis of a statistically significant association between age and CTS. However, as it has been mentioned in the results, further analysis has shown that age was strongly correlated with the cumulative exposure to keyboard strokes. We believe that the most interesting finding of the present study is that cumulative exposure to keyboard use as an independent predictor of the development of CTS, and a dose–response relationship have been documented. Our study has several limitations. One limitation is related to its cross-sectional design which does not allow us to conclude if the association between cumulative exposure to key-board use is of causative nature. The study included workers present when the study was performed, which implies a possible selection bias as is the case in all cross-sectional studies, especially if the study population was affected by high turn-over. It’s a limitation of our study that we don’t have data on actual turn-over of the staff. Moreover, despite of the satisfactory response rate (80 %) we were not able to collect information from non responders. In addition, the inclusion among cases of subjects who had been diagnosed or had surgery for CTS raises some issues which need further discussion. First, the association with cumulative keyboard use may have been confounded by the duration of employment, which is expected to correlate with both cumulative exposure and the likelihood of developing CTS, because the referral period increased with longer company seniority. Additionally, given that the time when CTS was diagnosed (case definition A) was not taken into account in the computation of cumulative keyboard use, relevant cumulative exposure could be overestimated for past CTS cases. Further, we didn’t control for possible confounding factors like anthropometric characteristics of the wrist, wrist position, use of wrist pad and type of keyboard [13-15]. Last, it’s a limitation that our study population was mainly consisted by females. Consequently, our results can be hardly extrapolated to male computer workers. Notwithstanding these limitations our study presents several advantages. A major limitation of the previous studies concerning the association between computer work and CTS is related to the assessment of the exposure to keyboard use which-in the most of the cases- was based on self-reports. Moreover, the cumulative exposure to key-board use was not taken into account regarding exposure assessment. On the contrary in the present study the exposure assessment was objective because the payroll system of the company under study was based on the records of daily keyboard strokes per employee. Another advantage of our study is that we have controlled for a variety of confounding factors like age, sex, BMI, personal medical history of disorders associated with the occurrence of CTS. Finally, we used two case definitions in order to verify the association between keyboard use and risk of CTS. Despite the fact that subjects belonging to both case definitions were examined by a physician, there is a difference between the two case definitions that deserves to be discussed. The first case definition was specific and restrictive because it included only cases with a history/surgery for CTS. On the contrary the second definition could be considered as more sensitive because it was more inclusive and included subjects that belonged to the first case definition plus cases identified through clinical examination).

## Conclusion

In conclusion, the present study indicated a possible association between cumulative exposure to keyboard strokes and the development of CTS after controlling for several confounding factors. In addition, a dose–response relationship between cumulative exposure to key-board strokes and CTS has been found. Cumulative exposure to key-board strokes would be taken into account as an exposure indicator regarding exposure assessment of computer workers. Further research is needed in order to test the results of the current study and assess causality between cumulative keyboard use and development of CTS.

## Competing interests

Authors declare that they don’t have any conflict of interests.

## Authors’ contributions

AE participated in the collection of data, study design and preparation of the manuscript. GR participated in study design, statistical analysis, preparation and revision of the manuscript. SV participated in preparation and revision of the manuscript. CK participated in the preparation and revision of the manuscript. CNM participated in the revision of the manuscript for important intellectual content. CH supervised study design, and statistical analysis. Also CH participated in the preparation and revision of the manuscript for important intellectual content. All authors read and approved the final manuscript.

## References

[B1] Dawson DM Hallett M Millender LH Entrapment neuropathies 1990 Boston: Little Brown and Co

[B2] Rempel D Evanoff B Amadio PC de Krom M Franklin J Franzbau A Consensus criteria for the classification of carpal tunnel syndrome in epidemiologic studies Am J Public Health 1988 88 1447 1451 9772842 10.2105/ajph.88.10.1447 PMC1508472

[B3] Palmer KT Haris EC Coggon D Carpal tunnel syndrome and its relation to occupation: a systematic literature review Occupational Med 2007 57 57 66 10.1093/occmed/kql125 17082517

[B4] Thomsen JF Gerr F Atroshi I Carpal tunnel syndrome and the use of computer mouse and keyboard: A systematic review BMC Musculoskelet Disord 2008 9 134 10.1186/1471-2474-9-134 18838001 PMC2569035

[B5] Rempel D Gerr F Intensive keyboard use and carpal tunnel syndrome: comment on the article by Atroshi et al Arthritis & Rheumatism 2008 58 1882 1883 18512800 10.1002/art.23509

[B6] Choi BCK Slopes of a receiver operating characteristic curve and likelihood ratios for a diagnostic test Am J Epidemiol 1998 148 1127 9850136 10.1093/oxfordjournals.aje.a009592

[B7] Graham B The Value Added by Electrodiagnostic Testing in the Diagnosis of Carpal Tunnel Syndrome J Bone Joint Surg Am 2008 90 2587 2593 10.2106/JBJS.G.01362 19047703

[B8] Van Rijn RM Huisstede BMA Koes BW Burdorf A Associations between work-related factors and the carpal tunnel syndrome.A systematic review Scand J Work Environ Health 2009 35 19 36 10.5271/sjweh.1306 19277433

[B9] Ali KM Sathiyasekaran BW Computer professionals and Carpal Tunnel Syndrome (CTS) Int J Occup Saf Ergon 2006 12 319 325 16984790 10.1080/10803548.2006.11076691

[B10] Atroshi I Gummesson C Ornstein E Johnsson R Ranstam J Carpal Tynnel syndrome and keyboard use at work. A population based study Arhritis & Rheumatism 2007 56 3620 3625 10.1002/art.22956 17968917

[B11] Andersen JH Thomsen JF Overgaard E Lassen CF Brandt LPA Vilstrup I Computer use and carpal tunnel syndrome.A 1- year Follow up Study JAMA 2003 289 2963 2969 10.1001/jama.289.22.2963 12799404

[B12] Maghsoudipour M Moghimi S Denghaan F Ralimpanah A Association of occupational and non occupational factors with the prevalence of work-related carpal tunnel syndrome J Occup Rehabil 2008 18 152 156 10.1007/s10926-008-9125-4 18418702

[B13] Nathan PA Keniston RC Carpal tunnel syndrome and its relation to general physical condition Hand Clin 1993 9 253 261 8509465

[B14] Marcus M Gerr F Monteilh C Ortiz DJ Gentry E Cohen S A prospective study of computer users: II. Postural risk factors for musculoskeletal symptoms and disorders Am J Ind Med 2002 41 236 249 10.1002/ajim.10067 11920967

[B15] Lassen CF Mikkelsen S Kryger AI Brandt LPA Overgaard E Thomsen JF Elbow and wrist/hand symptoms among 6,943 computer operators: A 1- year follow- up study (The NUDATA Study) Am J Ind Med 2004 46 521 533 10.1002/ajim.20081 15490472

